# Using a developed co-culture device to evaluate the proliferation of bone marrow stem cells by stimulation with platelet-rich plasma and electromagnetic field

**DOI:** 10.1186/s12891-023-07042-0

**Published:** 2023-12-05

**Authors:** Chia-Wei Chang, Chih-Chin Lee, Jen-Chung Liao

**Affiliations:** 1 Department of Orthopedic Surgery, Chang Gung Memorial Hospital, Keelung Branch, Bone and Joint Research Center, Chang Gung University, Taoyuan City, Taiwan; 2 Department of Orthopedics Surgery, Bone and Joint Research Center, Chang Gung Memorial Hospital, Chang Gung University, No._5, Fu-Shin Street, Kweishian, Taoyuan, 333 Taiwan

**Keywords:** Bone marrow stem cells, Platelet-rich plasma, Co-culture device, Electric field stimulation

## Abstract

**Backgrounds:**

Bone marrow stem cell can differentiate to osteoblast by growth factors, pulsed low-intensity ultrasound and electric magnetic field. In the research, bone marrow stem cells were cultured; bone marrow stem cells in culture can be stimulated by platelet-rich plasma and electric field.

**Methods:**

The culture well of the co-cultivation device has a radius of 7.5 mm and a depth of 7 mm. It is divided into two sub-chambers separated by a 3 mm high and 1 mm wide barrier. The bone marrow stem cells were seeded at a density of 2 × 10^4^ cells and the medium volume was 120μl. Platelet-rich plasma (PRP) or platelet-poor plasma (PPP) was added to the other sub-chamber at a volume of 10μl. The bone marrow stem cells were subjected to different electric fields (0 ~ 1 V/cm) at a frequency of 70 kHz for 60 min.

**Results:**

The highest osteogenic capacity of bone marrow stem cells was achieved by addition of PRP to electric field stimulation (0.25 V/cm) resulted in a proliferation rate of 599.78%. In electric field stimulation (0.75 V/cm) with PPP, the proliferation rate was only 10.46%.

**Conclusions:**

Bone marrow stem cell with PRP in the co-culture device combined with electric field at 0.25 V/cm strength significantly promoted the growth of bone marrow stem cells.

## Introduction

Surgeries for spinal arthrodesis or long bone nonunion often require bone grafts, which can come from autologous, allogeneic, or artificial sources. However, autologous bone grafting is limited by available bone mass and significant donor morbidity [1, 2], while allograft bone has limited osteoinductive properties, a high rate of false fusion, and a risk of disease transmission [3–5]. Although artificial bone substitutes such as Calcium Sulfate, Calcium Phosphate, or Hydroxyapatite are also used in spinal fusion surgery, they primarily act as osteoconductive agents {[6] ref}. In recent years, cell-based tissue engineering for bone substitutes has become increasingly attractive, as studies have shown that mesenchymal stem cells combined with various scaffolds can accelerate bone formation [7, 8]. Additionally, the enhanced transformation or osteogenesis of bone marrow stem cells into osteoblast-like cells has been linked to the induction of certain media, growth factors, cytokines, low-intensity ultrasound, hypoxia, and electromagnetic fields [9–14].

Platelet-rich plasma (PRP) is a platelet concentrate obtained by centrifugation of peripheral blood that contains various osteoinductive growth factors, such as transforming growth factor β1 (TGF-β1) and platelet-derived growth factor (PDGF) [15–17]. Although there are many studies investigating the efficacy of PRP in bone fusion, the results are inconsistent [18–20]. Some studies have demonstrated that concentrated platelet-rich fibrin gel is not effective for posterolateral fusion of the spine because of its tendency to drain [19], while others have revealed that PRP integrated with bone substitutes has positive ability to stimulate union in bone cavities [20]. In previous methods, the proliferation and ossification of bone marrow stem cells were investigated through electrical stimulation, or the ossification capacity of collagen sponge mixed with PRP was compared in long bone defects or spinal fusion regions [14, 17, 18, 20].

In this study, we developed a co-culture device to respectively seed bone marrow mesenchymal stem cells and apply PRP gel in different sub-chambers under the liked medium. In comparison to previous methods [14, 17, 18, 20], the key advantage of this co-culture device is that bone marrow stem cells can simultaneously receive growth factors released by PRP and undergo electric field stimulation. This specific co-culture device can be embedded with a pair of parallel plate electrodes that can stimulate bone marrow mesenchymal stem cells with an electric field. We aim to investigate the osteogenic response of bone marrow mesenchymal stem cells stimulated by the combination of PRP and electric fields.

## Materials and methods

The study received approval from the Institutional Animal Care and Use Committee of Chang Gung Memorial Hospital (Approval number: 2018121810). The study is reported in accordance with ARRIVE (Animal Research Reporting of In Vivo Experiments) guidelines.

### Isolation and culture of rabbit bone marrow stem cells

**Fig. 1:**
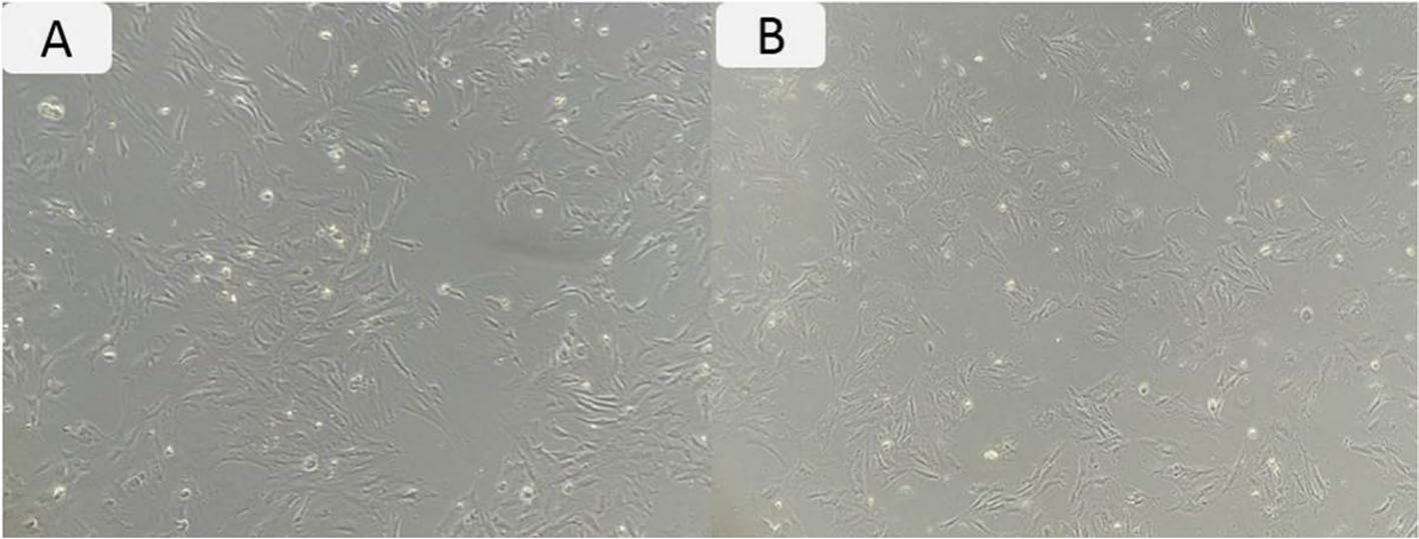
Image A shows the first-generation bone marrow stem cells isolated from the white rabbit cultured for four days. Image B shows the second-generation bone marrow stem cells isolated from the white rabbit cultured for three days

The New Zealand White Rabbits were given intramuscular injections of 17.5 mg/kg Zoletil (Virbac Laboratories, Carros, France) and 0.05 ml/kg Rompun (Bayer HealthCare, LLC) for anesthesia. The rabbits had their back hair shaved and were sterilized with iodine. A longitudinal incision was made in the skin and fascia on the iliac crest, and an 18G needle was used to create a bone window on the iliac crest. The bone marrow was extracted using an 18G syringe, and then the rabbits were sacrificed with ketamine (600 mg, IV) and their humerus and femur were harvested to isolate bone marrow stem cells (BMSC). The BMSC were washed with Dulbecco's Modified Eagle Medium (DMEM, Gibco) containing 1% Penicillin–Streptomycin (p/s, Gibco). The resulting cell suspension was centrifuged twice to remove impurities. After the two centrifugations, the resulting cell pellets were re-dissolved in DMEM and added to a 100 mm culture dish at an appropriate density for 4 days. The medium was then replaced to wash away residual blood cells. The BMSC were cultured and passaged in DMEM containing 20% Fetal Bovine Serum (FBS, Gibco) and 1% Penicillin–Streptomycin (P/S, Gibco) at 5% CO_2_ and 37 °C, as shown in Fig. 1. The medium was replaced twice a week to remove unattached cells and cellular waste products.

### Preparation of PRP and PPP

The rabbits were first anesthetized with an intramuscular injection of 17.5 mg/kg Zoletil (Virbac Laboratories, Carros, France) and 0.05 ml/kg Rompun (Bayer HealthCare, LLC). After the anesthesia, their ears were disinfected with iodine solution, and blood was collected from the ear arteries using a 22G needle. Approximately 40 mL of blood was collected from each rabbit using Vacutainer™ tubes containing Acid Citrate Dextrose (BD) for the preparation of platelet-rich plasma. The whole blood was then centrifuged at 1500 rpm for 10 min, and only the plasma was collected and centrifuged again at 3000 rpm for 10 min. The resulting supernatant was platelet-poor plasma (PPP), and the platelet-rich plasma (PRP) settled at the bottom of the centrifuge tube after resuspending the PPP. The amount of platelet in PRP was calculated by hematology analyzer.

### Design and manufacture of the co-culture device

The co-culture device has been designed to culture bone marrow stem cells and platelet-rich plasma (PRP) separately. The growth factors contained in the PRP are flowed into the bone marrow stem cell sub-chamber under the interconnected medium. Electric fields are conducted to stimulate bone marrow stem cells in this device. The culture well of the co-cultivation device has a radius of 7.5 mm and a depth of 7 mm. It is divided into two sub-chambers separated by a 3 mm high and 1 mm wide barrier.

**Fig. 2:**
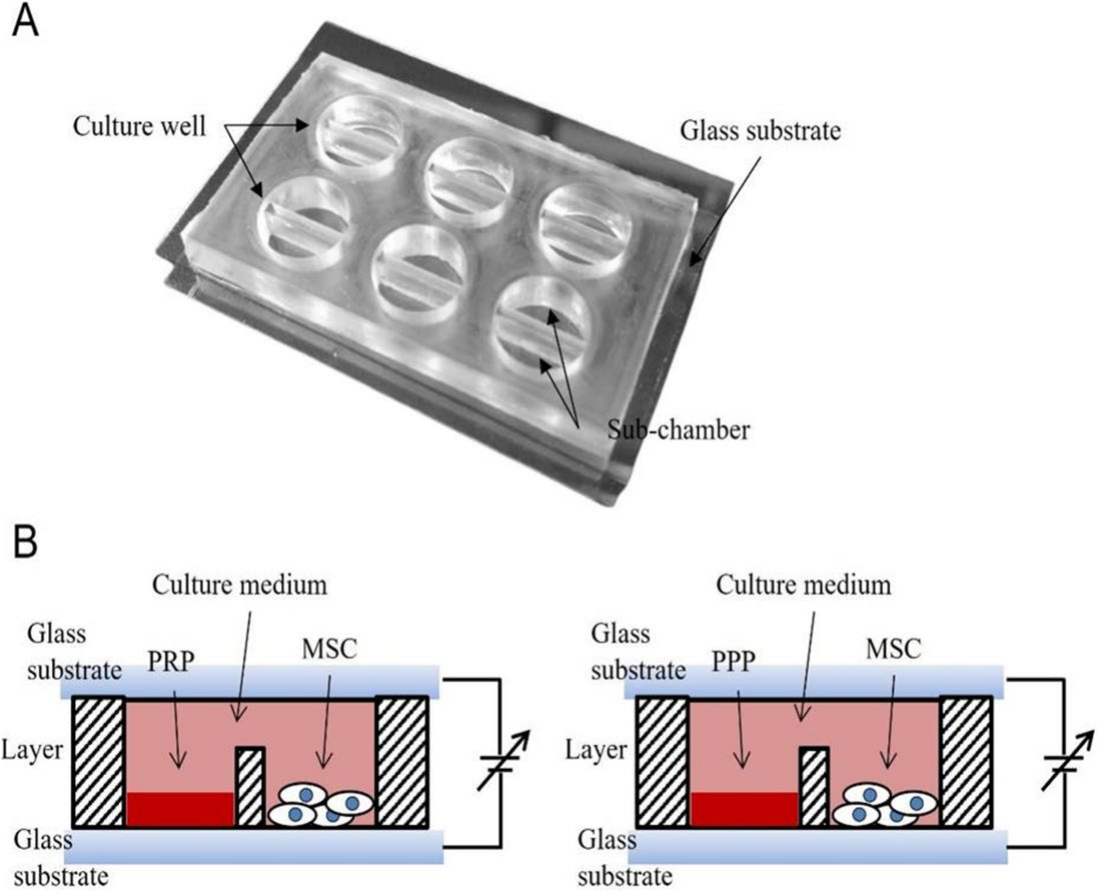
Design and tentative setup of the analysis of the proliferation of bone marrow stem cells by the stimulation of electric field and PRP. **A** Diagram of the co-cultivation device. **B** Tentative setup of bone marrow stem cells by the stimulation of electric field and PRP. PRP platelet-rich plasma, MSC marrow stem cell, PPP platelet-deficient plasma

The fabrication of the device is described briefly as follows: The co-culture device comprises a culture well, a glass substrate, and a polydimethylsiloxane (PDMS) layer. The PDMS layer was fabricated using a CNC engraving machine for polymethyl methacrylate (PMMA) machined negative pattern molds for culture wells. The PDMS layer is combined with an indium tin oxide (ITO) glass substrate to apply an electric field to the culture well. The glass substrate is also bonded simply to the PDMS layer, as shown in Fig. 2. The fabricated device was washed with phosphate-buffered saline and stored under UV light until the experiment was performed.

### Bone marrow stem cells stimulated by the electric field

The bone marrow stem cells were seeded at a density of 2 × 10^4^ cells and the medium volume was 120μl. Platelet-rich plasma (PRP) or platelet-poor plasma (PPP) was added to the other sub-chamber at a volume of 10μl. After the cells were allowed to attach overnight, 500μl of medium was added to connect the two sub-chambers across the partition wall in the co-culture device, as shown in Fig. 2. The bone marrow stem cells were cultured in a cell culture incubator for 7 days and were subjected to different electric fields (0 ~ 1 V/cm) using a waveform generator (DG1022, RIGOL Technologies) every day at a frequency of 70 kHz for 60 min. After the culture process, cell proliferation was evaluated using bioassays.

### Cell proliferation analysis

The proliferation of MSCs (bone marrow stem cells) following the combined stimulation of PRP and electric field was assessed using the Alamar Blue assay (Invitrogen) after 7 days. This assay can be detected using both fluorescence and visible light. Alamar Blue is initially dark and non-fluorescent. Upon action by NADH dehydrogenase in the mitochondria, Alamar Blue is reduced to pink, and the results of this reaction can be recorded by detecting fluorescence absorbance at 570 nm and 600 nm.

## Statistical analysis

The data was expressed as mean ± standard deviation (SD), and it was acquired through a minimum of three independent measurements. Analysis was performed employing the T-test, with statistical significance denoted as * for *p* < 0.05.

## Results

### Analysis of cell proliferation in different culture devices

**Fig. 3:**
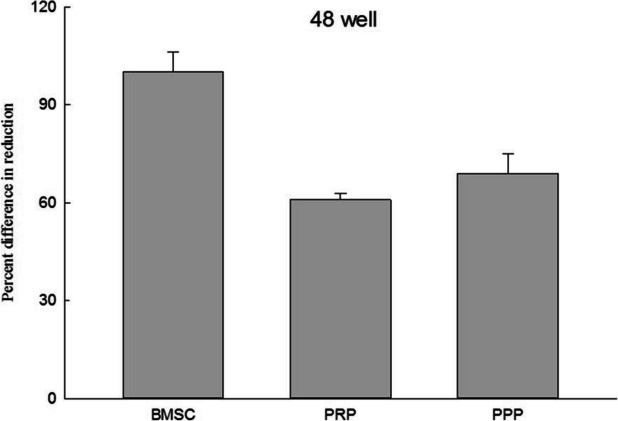
Graph of 48well cell proliferation analysis. This figure presents the cell proliferation ratio of BMSC only, combined with PRP, and combined with PPP in the 48-well plate. *n* = 3, BMSC bone marrow stem cell, PRP platelet-rich plasma, PPP platelet-deficient plasma

**Fig. 4:**
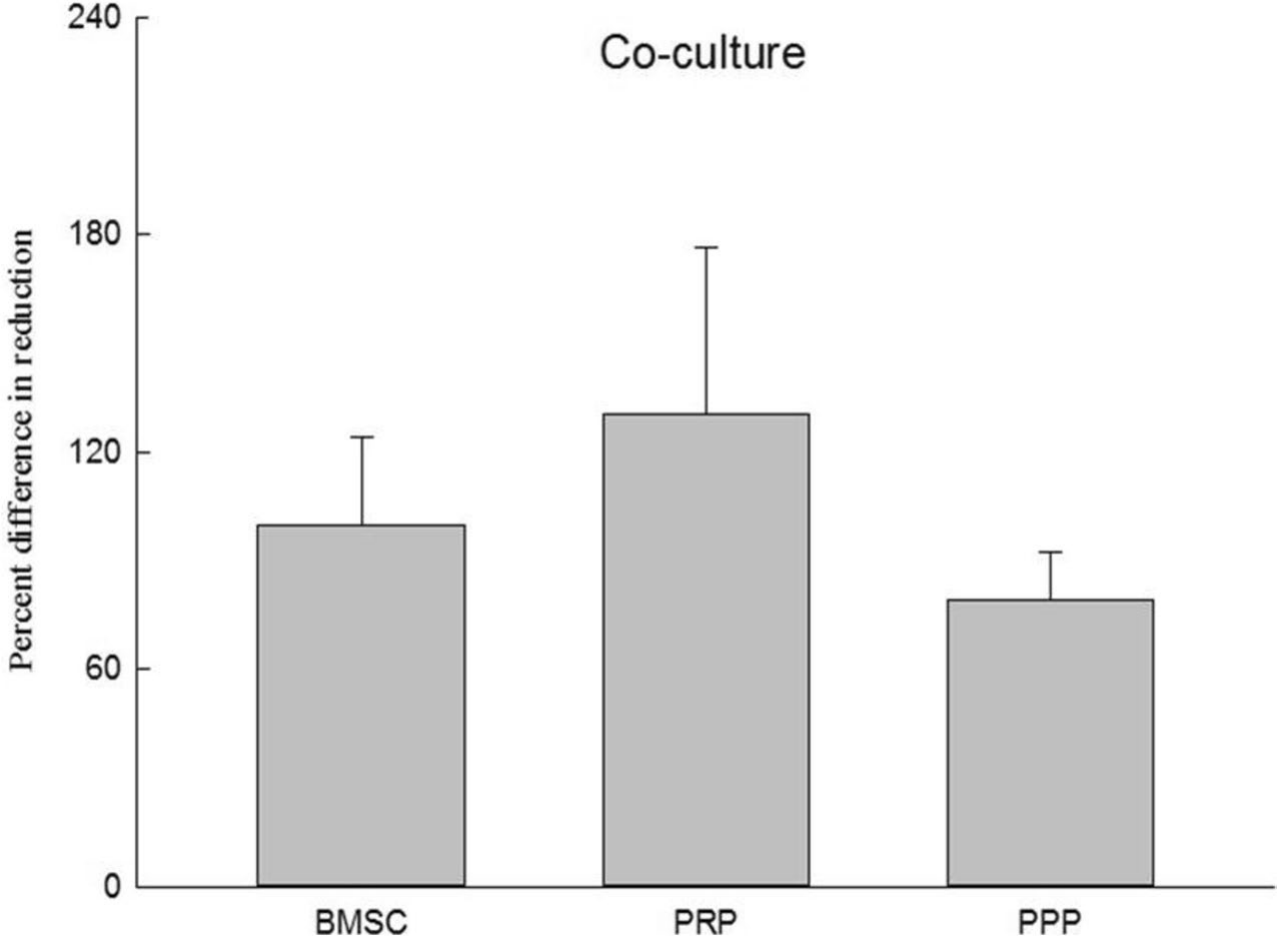
Graph of co-culture device cell proliferation analysis. This figure presents the cell proliferation ratio of BMSC only, combined with PRP, and combined with PPP in the co-culture device. *n* = 3, BMSC bone marrow stem cell, PRP platelet-rich plasma, PPP platelet-deficient plasma

The average platelet count in PRP is 2557.5 ± 761.56 × 10^3^/μl. A cell proliferation assay was conducted to evaluate how the growth of bone marrow stem cells was influenced by platelet-rich plasma (PRP) in various culture devices. The bone marrow stem cells were cultured up to the fourth passage (P4), and 2 × 10^4^ cells were seeded into 48 wells after washing them with 0.25% trypsin. Subsequently, 10μl of PRP, platelet-poor plasma (PPP), or medium were added to the wells based on the experimental groups. In the co-culture device, the bone marrow stem cells were also cultured up to the fourth passage (P4), washed with 0.25% trypsin, and 2 × 10^4^ cells were seeded into the sub-chamber. Then, 10μl of PRP, PPP, or medium were added to the subchambers according to the respective experimental groups. After the cells were attached, 520μl of medium was added to the subchambers to establish communication between them. The experiment was conducted for seven days. Following the seven-day period, Alamar Blue reagent was added to the cells, and after four hours of reaction time, the absorbance values (570 nm and 600 nm) were measured by the enzyme immunoassay reader (ELISA Reader). The obtained experimental values were then used to calculate and compare the results. The results showed that the average proliferation rate of bone marrow stem cells was 53.89% ± 2.53% with PRP and 61.05% ± 7.42% with PPP in the 48 wells. The average proliferation rate was 130.41% ± 65.34% with PRP and 79.03% ± 18.8% with PPP in the co-culture device. These findings are presented in Figs. 3 and 4, respectively.

### Analysis of cell proliferation stimulated by different electric fields

The bone marrow stem cells were cultured up to the fourth passage (P4) and washed with 0.25% trypsin. The cell count was 2 × 10^4^/120μl and seeded into a sub-chamber, while the other sub-chambers were filled with 10μl PRP, 10μl PPP, and 10μl medium, respectively. After allowing the cells to attach, 520μl of medium was added to the sub-chambers to establish communication between them. Each experimental group underwent five different electric field stimulations (0 V/cm, 0.25 V/cm, 0.56 V/cm, 0.75 V/cm, 1 V/cm) generated by a RIGOL Technologies waveform generator (model: DG1022), at a frequency of 70 kHz, for 60 min per day. The experiment was conducted for seven days.

**Fig. 5:**
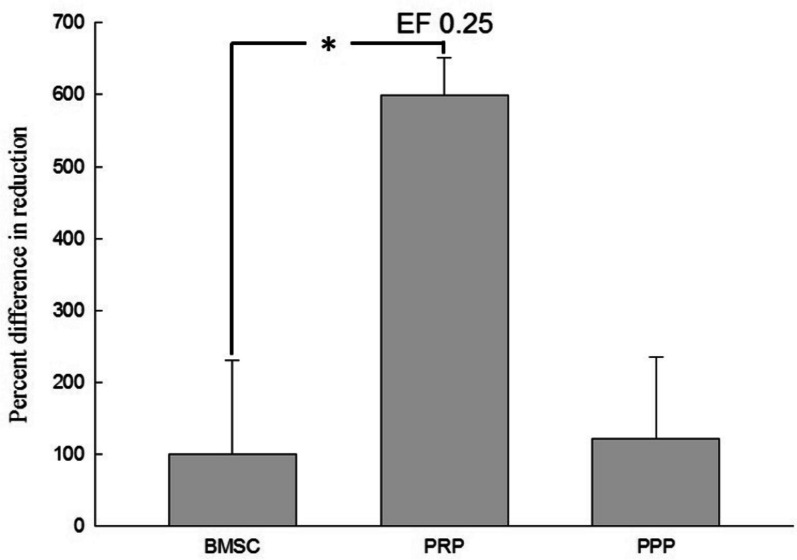
The data of electric field stimulated (0.25 V/cm) cell proliferation analysis. This figure presents the cell proliferation ratio of BMSC combined with electric field stimulation, PRP and electric field stimulation, and PPP and electric field stimulation in the co-culture device. It was significantly higher in the PRP group than in the BMSC. Electric field stimulation (0.25 V/cm) at 70 kHz for 60 min per day. *n* = 3, BMSC bone marrow stem cell, PRP platelet-rich plasma, PPP platelet-deficient plasma. *means a *p* value < 0.05

**Fig. 6:**
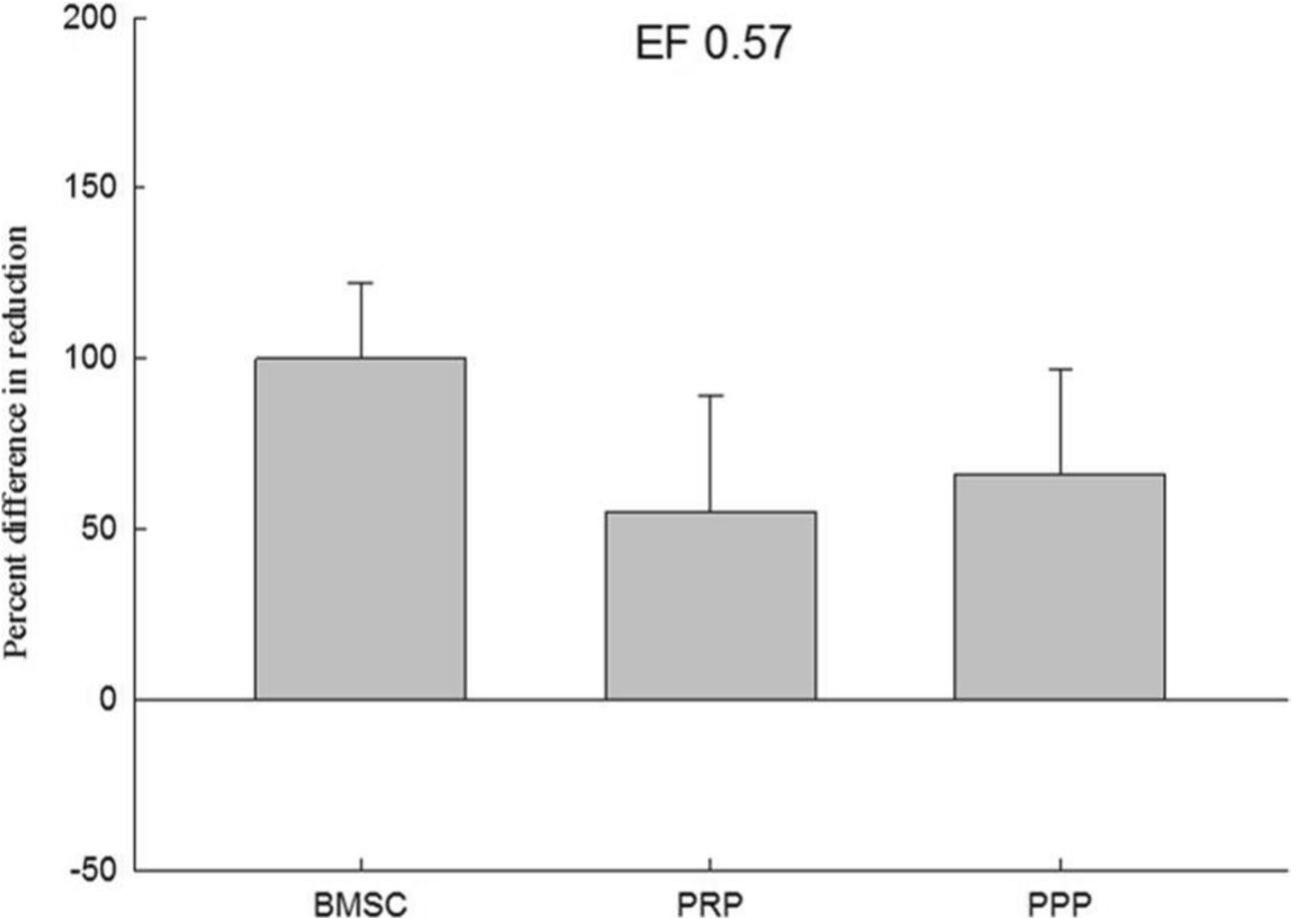
The data of electric field stimulated (0.57 V/cm) cell proliferation analysis. This figure presents the cell proliferation ratio of BMSC combined with electric field stimulation, PRP and electric field stimulation, and PPP and electric field stimulation in the co-culture device. Electric field stimulation (0.57 V/cm) at 70 kHz for 60 min per day. *n* = 3, BMSC bone marrow stem cell, PRP platelet-rich plasma, PPP platelet-deficient plasma

**Fig. 7:**
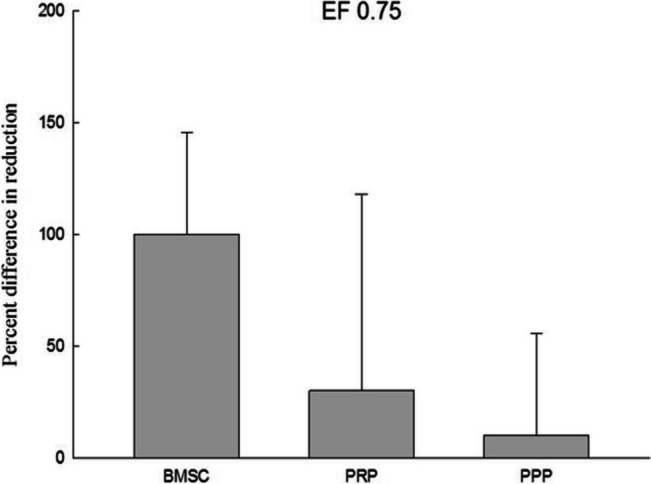
The data of electric field stimulated (0.75 V/cm) cell proliferation analysis. This figure presents the cell proliferation ratio of BMSC combined with electric field stimulation, PRP and electric field stimulation, and PPP and electric field stimulation in the co-culture device. Electric field stimulation (0.75 V/cm) at 70 kHz for 60 min per day. *n* = 3, BMSC bone marrow stem cell, PRP platelet-rich plasma, PPP platelet-deficient plasma

**Fig. 8:**
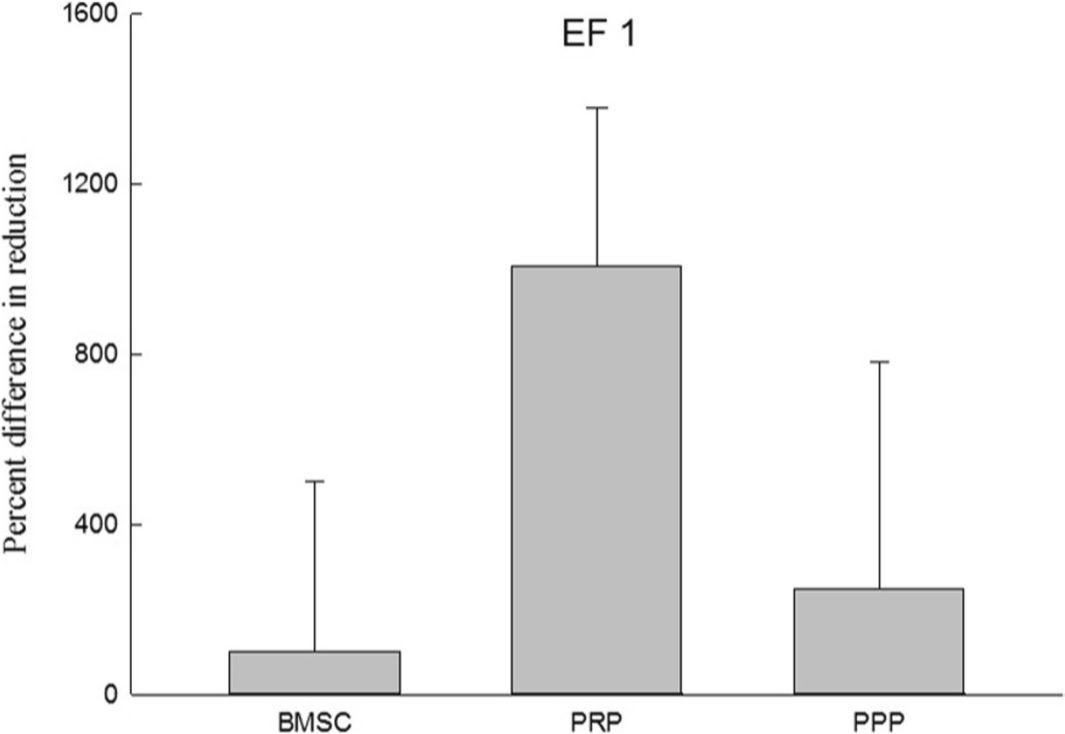
The data of electric field stimulated (1 V/cm) cell proliferation analysis. This figure presents the cell proliferation ratio of BMSC combined with electric field stimulation, PRP and electric field stimulation, and PPP and electric field stimulation in the co-culture device. Electric field stimulation (1 V/cm) at 70 kHz for 60 min per day. *n* = 3, BMSC bone marrow stem cell, PRP platelet-rich plasma, PPP platelet-deficient plasma

After seven days, 1/10 volume of Alamar Blue reagent was added, and the reaction was allowed to proceed for four hours. The absorbance values (570 nm and 600 nm) were then read using an enzyme immunoassay reader (ELISA Reader), and the experimental values were calculated accordingly. The results were compared and shown in Figs. 5, 6, 7, and 8. The average proliferation rate of bone marrow stem cells in each experimental group was determined under different electric field stimulations. The addition of PRP to electric field stimulation (0.25 V/cm) resulted in significant proliferation rate of 599.78% ± 72.3% (*p* < 0.05), while the addition of PPP resulted in a rate of 121.90% ± 159.65%. Adding PRP to electric field stimulation (0.57 V/cm) led to a rate of 54.94% ± 48.2%, while adding PPP led to a rate of 66.13% ± 43.26%. In electric field stimulation (0.75 V/cm) with PRP, the proliferation rate was 30.25% ± 124.25%, while it was 10.46% ± 64.47% with PPP. Finally, adding PRP to electric field stimulation (1 V/cm) resulted in a rate of 1007.65% ± 525.15%, while adding PPP led to a rate of 246.86% ± 759.21%.

## Discussion

The earliest known report on the osteogenic ability of PRP in an in vivo bone fusion model dates back almost 25 years. This report described the use of autologous fibrin adhesion to stimulate early bone consolidation of autogenous cancellous bone during mandibular continuity reconstruction [21]. Subsequently, Li et al. conducted the first study on PRP in a lumbar spinal fusion model in 2004, which showed that combining PRP with beta tricalcium phosphate granules resulted in only partial union in a lumbar interbody fusion on a pig [22]. Similarly, Cinotti et al. reported that PRP was ineffective in promoting new bone formation and vascularization in a rabbit lumbar posterolateral lumbar fusion model [23].

Research has shown that adding pure PRP to autologous bone in a posterior lumbar interbody fusion does not significantly improve outcomes compared to using autologous bone alone [24]. In order to achieve successful bone union or spinal arthrodesis with PRP, it is recommended to combine it with bone substitutes such as collagen, β-tricalcium phosphate (β-TCP), or hydroxyapatite (HA), which has been shown to result in a 60% fusion rate in a rabbit spinal arthrodesis model [25]. The concentration of platelets in PRP is also an important factor in determining its effectiveness for promoting bone regeneration. Weibrich et al. found that platelet concentrations in PRP within a specific range, typically between 2 and 6 times higher than the concentration of platelets in whole blood, can have a positive effect on bone formation [26].

The use of PRP in osteogenic abilities has yielded inconsistent results due to the concentration of platelets present. Lower concentrations have limited effects on stimulating bone formation, while highly concentrated PRP may have inhibitory and cytotoxic effects on osteoblast activity. In this study, the comparison of cell growth in different culture devices was investigated. When adding growth factors to culture plates, it is typically done directly. However, when growth factors such as PRP are not pure liquids, direct addition may cover the surface of the cells, affecting nutrient absorption and reducing the oxygen exchange rate of the cells, thereby hindering cell growth. The current study found that the average proliferation rate of bone marrow stem cells was 61.05% ± 7.42% when PRP was added directly to a 48-well culture device, but increased to 130.41% ± 65.34% when PRP was added to a co-culture device. Directly adding PRP glue to the 48-well device did not promote cell growth. However, using a co-culture device to separate the PRP glue and relying on the growth factors released from it to move to the sub-chamber where the cells are located via a concentration gradient led to an increase in cell growth.

External physical stimuli have been found to have positive effects on osteogenesis by enhancing the synthesis of extracellular matrix components and cytokines for cell proliferation. Various external physical stimuli, including external mechanical strains, low intensity pulsed ultrasound, electromagnetic stimulation, and direct-current electric stimulation, have been studied in this regard [27–30]. Intermediate frequency (kHz—MHz) DC electric fields have been reported to stimulate vascular endothelial cells to induce angiogenic responses [31]. In this study, different electric field strengths were compared to evaluate their effects on the proliferation of bone marrow stem cells added with PRP. It was found that at EF 0.25 V/cm and EF 1 V/cm, the average proliferation rate of bone marrow stem cells added with PRP was 599.78% ± 72.3% (*p* < 0.05) and 1007.65% ± 525.15%, respectively. On the other hand, the average proliferation rate of bone marrow stem cells added with PRP without electric field was 130.41% ± 65.34%, indicating that the co-culture device and these two electric field strengths can significantly promote the growth of bone marrow stem cells. Studies by Mobini et al. and Eischen-Loges et al. have also demonstrated the effects of electrical stimulation on rat bone marrow stem cells, showing that the treatment of rat bone marrow stem cells with osteoinductive factors and culture medium and giving 100 mV/mm electrical stimulation led to obvious growth and ossification phenomena [32, 33]. Additionally, electric field stimulation has been found to enhance neurite growth. Wood and Willits observed that short-term direct current stimulation with an electric field strength of 25 V/m for 10 min could stimulate neurite growth and growth rate for up to 48 h after stimulation [34].

## Conclusion

The results of the comparison of different electric field stimulations demonstrate that at EF 0.25 V/cm and EF 1 V/cm, the average proliferation rate of bone marrow stem cells increased by 599.78% ± 72.3% (*p* < 0.05) and 1007.65% ± 525.15%, respectively, when added with platelet-rich plasma (PRP), compared to the control group without electric field stimulation. Interestingly, the average proliferation rate of bone marrow stem cells added with PRP without electric field stimulation was only 130.41%, indicating that the co-culture device combined with these two electric field strengths significantly promoted the growth of bone marrow stem cells.

## Data Availability

The datasets generated during the current study are not publicly available due to our institute policy but are available from the corresponding author on reasonable request.
